# American Dental Association

**Published:** 1891-01

**Authors:** 


### Reports of Society Meetings.

### AMERICAN DENTAL ASSOCIATION.—THIRTIETH ANNUAL MEETING, EXCELSIOR SPRINGS, MO., AUGUST 5 TO 8, 1890.

(Continued from vol. xi., page 755.)

[Owing to an error in transcribing, the following remarks of Dr. Allport were accidentally omitted in the December issue, and should have followed Dr. Storey, p. 751.—Ed.]

*Dr. Allport.*—My friend, Dr. Storey, says that a diploma from any dental college should be a passport to practise dentistry anywhere. So it should, and were all our dental colleges satisfactory, it would be. But many of them are not, and we know it.

We know, too, that a large majority of the diplomas that have been issued by this class of colleges are no more an indication of their holders' qualification to practise dentistry than would be so many pieces of brown paper. And it is well known, too, that many of the teachers in these colleges are not qualified to properly practise what they assume to teach. It is necessary, therefore, that we have these State Dental Examining Boards, whose duty it is made to inquire into the quality of teaching done in our respective colleges, and determine as to the significance and value of the diplomas that they issue, as well as to the qualifications of those not graduates who propose to enter upon the practice of dentistry in our respective States.

Were it not for these boards the diplomas of one college would be of the same legal significance as another. Take, for instance, the State of Illinois. In the city of Chicago we have between thirty and forty regularly incorporated dental colleges.

*Dr. Baldwin.*—Twenty.

*Dr. Allport.*—We will say twenty then. According to the laws of our State, every one of these colleges has the same legal right to issue diplomas as has the Harvard, the University of Pennsylvania, the Chicago Dental College, or the University Dental College of Chicago, and but for the power given to our State Dental Boards to determine as to the respectability of these respective colleges, the diplomas of any of them would give their holders the legal right to practise dentistry in the State of Illinois.

Under the general incorporate law of Illinois the requirements to obtain a charter for a dental college, with the right to issue diplomas of qualifications to practise dentistry, are for these men to band themselves together and send their dollars to our secretary of State and ask permission to incorporate and organize a dental college. A permit will be returned, no matter whether the proposed incorporators know anything about dentistry or not.

Our State Board is the only protection that the people have to secure them against graduates of these dishonest concerns, and honest colleges should regard our State Dental Boards as their best friends.

Could we have a unification of the dental laws throughout the different States, as has been suggested, it would no doubt be a good thing, but so long as each State acts independently of the others, I do not see how this can be secured through the State legislatures, nor could it be done by an act of Congress, for the Constitution of the United States gives to each State the right to regulate all such matters as it deems fit. But our National Association of Dental Examining Boards can, by agreement between the various State Boards, make themselves almost as powerful and useful as would an act of Congress or a unification of our State laws upon the subject.

I can see, as Dr. Brown says, that in some cases it would be a seeming hardship for well-known qualified practitioners to be obliged to be examined by a State Board before they could practise in a particular State. But for the good of all such a law is not without merit, for no one should be allowed to practise in any State unless the State Board was satisfied they were qualified to do so. But no sensible board would deem it necessary to enter into a critical examination of Dr. Atkinson or Dr. Storey before allowing them to practise within their State. The law only requires that the State Board shall be satisfied that applicants are qualified to practise, and no need for an examination exists when the applicant is known to be qualified. It is not always the letter but the spirit of the law that should be complied with.

In reference to what Dr. Noble has said about urging our representatives to vote for the bill now pending before Congress, to regulate the practice of dentistry in the District of Columbia, it seems to me it would be a good thing to do, and I would be willing to do what I could in that direction. But it has occurred to me that a better thing would be for this Association to pass a resolution requesting Congress to pass a bill now pending before it on this subject, and that a resolution of this kind from the American Dental Association could but be treated with respect and consideration.

*Second Day's Proceedings.—Evening Session.*

The evening session, second day's proceedings, was called to order by President Foster at 8 P.M.

A communication was read from the National Board of Dental Examiners, calling attention to the fact that considerable litigation had been going on in New Hampshire, and that the dental law in that State had been declared unconstitutional by the Supreme Court. It was also stated that the attempts to sustain the law in the lower courts had been feeble, owing to the lack of funds.

A letter of regret was read from Dr. W. H. Dwinelle, of New York.

Dr. Patterson, of Kansas City, Mo., Dr. Atkinson, of New York, and Dr. Goddard, of San Francisco, Cal., were appointed a committee of three to represent the Association upon the National Board of Dental Examiners.

A resolution was passed requesting Congress to pass the law regulating the practice of dentistry in the District of Columbia.

Telegrams of condolence were sent to Dr. W. H. Morgan, of Nashville, Tenn., and Dr. E. T. Darby, of Philadelphia, absent from the convention on account of illness.

A cablegram of congratulations was sent to the International Medical Congress in session at Berlin.

Dr. Truman offered a resolution to the effect that the lantern exhibits be limited to one hour, to be divided between the two lecturers.

The resolution was carried.

Dr. Peirce, chairman of Section 2, then stated that the section had no further report to make. He hoped that some action would be taken by the Association looking to the establishment of a degree higher than that of D.D.S. It would be an additional encouragement to students and young men who are ambitious in the struggle for scientific attainment, and would accomplish much which would be of advantage to the profession.

Dr. Patterson introduced a resolution, asking that delegates coming from local societies be invited and urged to bring with them interesting and original matters and reports that might be of interest to the members of the Association. The resolution contemplated the selection of one of the delegates of the local society to select the material in order that it might be placed in the best possible manner before the American Dental Association. It was explained that the resolution was a little premature to the plans of the committees, and it was withdrawn by request.

Dr. Smith said that he was interested in the plan of a higher degree than that of D.D.S. He thought that it might be possible to establish an academy of dental science, and that, by due diligence in the choice of the instructors and the graduates, a school of this character might become a great factor in dentistry.

Section 2 was then passed and Section 3 was called. This is the section of Operative Dentistry, E. T. Darby, chairman, N. S. Hoff, secretary. Dr. Crouse, of Chicago, in the absence of Chairman Darby, stated that two reports had been presented to the section. These reports, he said, had been condensed, and would be read by Dr. Baldwin, of Chicago. The report of Dr. Baldwin was as follows:

Two papers were referred to this section by the other sections, and to the writer was referred the task of presenting an epitome of the salient points of each.

The first is upon medicated oxyphosphate fillings, by Dr. Charles B. Atkinson, of New York. In this paper he advises, after an experience of two years in capping exposed pulps, the admixture of various medicaments with the filling-material. The medicaments used are, 1, creosote and oil of cloves; 2, eugenol; 3, deliquesced carbolic acid; 4, oil of cinnamon; 5, oil of cloves; 6, creosote pure; 7, creosote, oil of cloves, and iodoform; 8, creolin; 9, campho-phénique; 10, potassium chlorate powder; 11, salicylic acid; 12, camphor pulvis; 13, stick sulphur pulvis; 14, iodoform; 15, oil of winter-green.

The principle of medicinal action of the mixture is the principle that, upon the crystallization of the oxyphosphate, no further change takes place, hence the remedy may exert some of its remedial action. He employs mostly the first mixture (creosote and oil of cloves). When the medicament is a liquid, he adds about an equal quantity of medicament and phosphoric acid, and when a solid, about equal parts of medicament and oxide. These proportions may be varied as required. He believes that in this way the remedial agent is constantly exerting its effect upon the walls of the cavity, thus resisting germ-action, and also those additions producing increased hardness and increased resistance to thermal changes. He, from his tests, thinks that the first has been fairly tested, and that they are about equal in durability, manipulation, and time of setting in about ten minutes. He has made no effort to control color. His practice is to freely excavate even to the surface of the pulp and remove almost all of the infected matter in the cavity. He employs this system further in retaining inlays, straightening pyorrhœa cases,—*i.e.*, in setting isolated blocks or single teeth in rubber or gold dentures. The writer always, in capping pulps, covers the exposure with the medicated cement.

The second paper is entitled "A Few Words on Corrective Preservative Obtundent Treatment of the Teeth." It is by Dr. J. L. Williams, of Boston, Mass., in which he says that as early as April, 1856, in the *American Journal of Dental Science*, he gave his treatment of teeth where pulps were endangered but not exposed, the main idea being the saturation and the sealing of the cavity with non-irritant corrective and antiseptic applications, unsealing and repeating when necessary. Mild applications were found to be more effective than stronger. His experience to this time bears out these principles, recognizing that nature only requires mild help in removing diseased conditions, and that the reparative processes are hers.

The writer maintains that the treatment should be a gradual preservative treatment. At the time mentioned (1856) known antiseptics were comparatively few. The writer then used oxide of zinc to stiffen the gutta-percha, which has since then become well known. As a single obtundent he often uses a solution of calcium in checking fermentative action. However, the great object should be not to see how much nature will bear but to know how she works and to assist her. The writer claims in the paper, historically, in relation to the origin of two things, first, the systematized plan favoring the deposit of secondary dentine; second, the first use of oxide of zinc mixed with gutta-percha for a plastic stopping. Hill's stopping was a prior and a patented preparation, having no oxide of zinc and no sulphate of lime to stiffen it.

Dr. N. S. Hoff then presented a brief report of improvements introduced during the year. Two additional materials for filling roots have been suggested,—red cedar, saturated with paraffine, by Dr. James H. Beebe, and beeswax, by Dr. B. F. Arrington. Dr. Taylor, of Hartford, suggests dropping chloroform in the root-canal and then dissolving gutta-percha in it, then forcing a solid cone into the root.

A set of needles, by Dr. W. S. How, for use in making dressings.

For the treatment of sensitive dentine the actual cautery has been recommended by Dr. J. W. Holt.

Dr. R. S. Williams has brought out a new form of crystalloid gold.

Dr. Geo. H. Wiegant has devised a set of diamond trephines for cutting out sections of porcelain.

Dr. E. C. Moore has devised a set of metallic racks for keeping instruments.

Dr. Letord, a double-faced mouth-mirror.

Dr. H. H. Knapp, an apparatus for holding a mouth-mirror and large magnifier in place with rubber dam, etc.

Dr. John L. Gish has invented an improved rhœostat for controlling the electric current.

Dr. J. W. Ivory has introduced a pair of molar clamps for the third molar, also a nerve-broach, spiral in form, acting on the principle of the cork-screw.

The S. S. White Dental Manufacturing Company have introduced a number of improvements.

Hood & Reynolds have invented a new dental chair.

Dr. Peirce then addressed the association upon the subject of peroxide of hydrogen. He stated that this material was familiar to all of the members, because of the uses to which it had been applied in the cleansing of pus cavities. It has also been widely used in removing stains from teeth. It occurred to him that the action of the agent on the tooth-structure had never been thoroughly tested. July 7 he placed a bicuspid in a drachm and a half of the liquid. It remained there twenty-four hours. The doctor then discovered that lime had been precipitated in the bottom of the vessel. He again placed the tooth in the liquid, and kept it there until the surface was removed and the dentine was reached. The action on the dentine was much slower than on the cementum. There was no softening of the material of the tooth. Dr. Peirce stated that he had brought the flask containing the liquid with him, but the deposits at the bottom had been disturbed in transit. He thought that the destruction of the organic matter had been proved by the experiment. The tooth had been reduced to two-thirds its size in seventeen days.

Dr. Brophy stated that peroxide of hydrogen is decidedly acid, through the addition of sulphuric acid, and that it should be neutralized before using.

Dr. Rhein said that he had tried this experiment with peroxide of hydrogen on lime deposits out of the mouth, and he had found a very large precipitation formed in the manner suggested by Dr. Peirce. He had not finished his experiments with the calcareous formation, but he believed that peroxide of hydrogen had a deleterious effect.

Dr. Harlan said that the question of the action of peroxide of hydrogen had received too little attention from the profession, and that this specimen of Dr. Peirce's presents a decidedly acid reaction. When pure it should be neutral. Then there would be no ill effects upon the teeth. He advised the members of the Association to get the acid that is perfectly neutral. He then referred to the use of vulcanized rubber as an inlay. He remarked that last year, while in Europe, he noticed some beautiful specimens at Naples. He thought that it offered to the profession a cheap and serviceable filling.

Dr. Ames thought Professor Peirce must be in error. While in the solution of dentine by chromic acid there is no precipitate because the chromate of calcium formed is soluble, if the solvent be sulphuric acid contained in peroxide of hydrogen, the sulphate of calcium formed would give a precipitate.

Dr. Stubblefield, referring to the experiment made by Dr. Peirce, said that the ordinary peroxide of hydrogen is derived by the action of hydrochloric acid on barium dioxide. If there exist unequal portions of these two ingredients there will remain some of the hydrochloric acid, and the acid reaction observed will be found to be due to this. Sulphuric acid is never added to prevent the hydrogen peroxide from loss of the combined oxygen.

It seemed to him that to dilute hydrochloric acid with pumice would make a better cleanser than peroxide and pumice, because the rapid evolution of oxygen when this peroxide is exposed to the air leaves only $H_2O$,—water.

Dr. Peirce inquired, "If the tooth be placed in hydrochloric acid, cannot organic material be found on the surface of the tooth?"

*Dr. Stubblefield.*—Yes, I would find the basis substance of the tooth.

Dr. Brophy replied that he did not come to the conclusion through examination, but that he had it on the word of a chemist to be relied upon.

Dr. Conrad said that Dr. Fisher had made the statement, at the Chicago meeting, that sulphuric acid is placed in hydrogen peroxide. He said that St. Louis and Chicago manufacturers admitted this.

Dr. Harlan said that some of the chemists admit that sulphuric acid is placed in hydrogen peroxide. When lecturing to students he told them that they must procure hydrogen peroxide without an acid reaction.

Dr. Atkinson said that he had been in the habit of calling hydrogen peroxide his washerwoman, but he insisted that great care should be taken in using it. He had brought samples of hydrogen peroxide with him, but the express companies had delayed in delivering the packages. He had been disappointed in his own experiments, and he desired to enter a protest against calling careless experiments scientific.

Dr. Ottofy said that he had used iodoform and eucalyptol with good effect, and he had reason to believe that it was a germicide.

Dr. Storey then related his experiments in trying to save pulps. He related several cases that had proved perfect failures in spite of the greatest care exercised. There would be no discoloration, though the tooth would ache and the patient would remain unhappy. He then went on to say that he didn't use any medicines. When he started in to practise medicine he had had saddle-bags and a great coat filled with medicines. He had found oxychloride of zinc excellent for filling the roots of teeth, and when one thing gave him satisfaction he was not disposed to run after anything else unless convinced that it was an improvement. He had found trouble in operating with all of the small instruments introduced to the profession. They were said to be of use in small cavities, but if they were small he was of the opinion that the trouble would be small. He had used peroxide of hydrogen, but it did not meet the conditions. He had then reached the conclusion that he hadn't the ability to use it, and wisely concluded to stick to materials that he did have the sense to use.

Dr. Ames asked Dr. Peirce if he had tested the solution, after it had been used, to see if it had all of the properties of peroxide of hydrogen.

Dr. Peirce replied that he had not.

Dr. Brown advised the members of the American Dental Association not to let any one scare them away from saving of pulps. He said that he had known South Sea Island asbestos to be incorporated with oxyphosphates with excellent results. Asbestos is a non-conductor. There are a thousand kinds of asbestos, but few are good for purposes of this nature. He held that it could be used as a non-conductor under gold filling. He had dreaded very much thermal changes, but his patients had not complained of any additional trouble after the teeth had been filled.

Dr. Swasey said that he would like to mention the matter of an inlay already mentioned by Dr. Harlan. He said that white rubber wears out, not so fast as gutta-percha, but still it wears out rapidly. The inlay that he used now he made from gold, and within the last six months he had been taking impressions from ribbon gold. He placed the gold over the cavity and burnished it as well as possible to the walls. Twenty-carat gold should then be placed in strips in the shell formed and melted there. The inlay should then be tried in the cavity and filled.

Dr. Harlan then gave the tests that had been made with the peroxide of hydrogen furnished by Dr. Peirce. All indicated the presence of an acid.

Dr. Smith remarked that he would like to say something about the preservation of the pulps. He had been taught by histology the proper location of the pulp. From this locality extend the tubuli. These tubuli are filled with an organic substance, and whenever decay occurs there is a destruction of the tubuli. The branches and the foliage of the plant depend upon the harmony of all the parts. Nature is symphonic in its action. It isn't always chemical influences that will destroy pulps. It is suction, the drawing towards the periphery, that kills. Strip a man of his limbs, and he may live, but the harmony is gone forever. You must preserve the harmony. The brain is located in the head, but if you amputate an arm you take away a part of the brain. It is the subtle influence that destroys and kills. My prayer has always been, "Save the pulps." I have made successes in this respect and I have made signal failures. I accord it to the reasons just mentioned, a failure or a success in complying with the conditions.

Dr. Goddard said that he was not here to speak of implantation, but to answer an inquiry that had been made. He held that it is always easy to find a suitable root for any case of implantation by making a groove between the neck of the tooth and the neck of the root. In this manner it is possible to obtain a perfect groove, and it cannot be obtained in any other way.

Dr. Storey thanked Dr. Smith for his explanation. In almost every instance where the pulp had died he had found that something had been put into the tooth to allay pain when filling.

Dr. Crouse said that straws tell in dentistry. His experience did not always agree with others. He had known certain men who never had had any success in capping pulps. He could not tell whether it was a lack of accuracy, carelessness, or something else. But there must be a reason for it. He had participated in discussions before, lost faith in some good old remedy, and had gone back home to try something else, and then he had been driven back to the old remedies. But he still believed that a tooth was better capped than out of the head.

Dr. Storey replied that he lived in Texas, while Dr. Crouse lived in Chicago. He said that there was a funeral in his office three or four times a day. He killed all of the pulps, and he believed that he was doing a good work in ending them. He said that he did not practise dentistry for the purpose of giving himself and his patrons trouble. He added that his opponents had fought him at Dallas and they had fought him all over the State, but he still insisted that he was correct. To save a pulp for two or three years might be a good practice for Dr. Crouse, but it wasn't a good practice for Dr. Storey. He could account for the differences in the results by the differences in the climate if upon no other grounds.

Dr. Crawford was glad that the discussion had taken a practical turn. He said that the members of the profession had failed to take into consideration the different diagnoses made. The extremes were represented by Dr. Crouse and Dr. Storey. He preferred to occupy a medium ground. He held to the principle that when a smaller organ is affected it seldom affects a larger organ. It works the other way, from the larger to the smaller. He always tried to save the pulp. If he failed to relieve the extraordinary sensibility, then he returned to the treatment advocated by Dr. Storey.

Dr. Baldwin stated that in the general practice of surgery it was expected that some allowances should be made for climatic conditions. But allowances were to be made for personal conditions as well. The gentlemen who have been discussing the two sides of the question might not be so far apart as they seemed to think. He had seen teeth where the pulp had been saved, but upon examination he had found the pulp dead. It had died a painless death. It was dormant, if the expression would be allowed for the moment. The explanation might be found in the changed current of nourishment. The increased nourishment renders the parts hypersensitive. Then there is a difference in the characteristics of the patient. It is better to make an attempt to save the teeth of a healthy young person than the teeth of an old or diseased person.

Dr. Rhein wanted it understood that every day that the pulp remains in the tooth so much the better for the tooth in its after development.

Dr. Conrad said that he deemed it eminently proper to attempt the capping of pulps in young subjects. He believed that climate had a great deal to do with success or failure in certain lines of treatment. He would rarely attempt to cap an exposed pulp in the tooth of an old person.

The following-named dentists were selected to act as a commission to arrange for the dental congress to be held at Chicago, in connection with the World's Fair, in 1893: Dr. J. Y. Crawford, Nashville, Tenn.; Dr. John Storey, Dallas, Tex.; Dr. L. D. Carpenter, Atlanta, Ga.; Dr. Barton, Paris, Tex.; Dr. C. E. Stockton, Newark, N. J.; Dr. Taft, Cincinnati, O.; Dr. L. D. Shepard, Boston, Mass.; Dr. W. W. Walker, New York, N. Y.; Dr. Noble, Washington, D. C.; Dr. A. O. Hunt, Iowa City, Iowa; Dr. Marshall, Chicago, Ills.; Dr. McElhaney, Augusta, Ga.; Dr. M. W. Foster, Baltimore, Md.; Dr. H. J. McKellops, St. Louis, Mo.; and Dr. A. W. Harlan, Chicago, Ills.

The National Association of Dental Examiners at its session today elected the following officers: Dr. C. R. E. Koch, of Chicago, Ills., president; Dr. L. C. Wasson, of Topeka, Kan., vice-president; Dr. J. H. Martindale, of Minneapolis, Minn., secretary and treasurer.

(To be continued.)

